# Impact of Adjuvant Radiotherapy on Survival Outcomes in Intermediate-Risk, Early-Stage Cervical Cancer: Analyses Regarding Surgical Approach of Radical Hysterectomy

**DOI:** 10.3390/jcm9113545

**Published:** 2020-11-03

**Authors:** Se Ik Kim, Tae Hun Kim, Maria Lee, Hee Seung Kim, Hyun Hoon Chung, Taek Sang Lee, Hye Won Jeon, Jae-Weon Kim, Noh Hyun Park, Yong Sang Song

**Affiliations:** 1 Department of Obstetrics and Gynecology, Seoul National University College of Medicine, Seoul 03080, Korea; seikky@naver.com (S.I.K.); marialeemd@gmail.com (M.L.); bboddi0311@gmail.com (H.S.K.); chhkmj1@snu.ac.kr (H.H.C.); kjwksh@snu.ac.kr (J.-W.K.); pnhkhr@snu.ac.kr (N.H.P.); 2 Seoul National University Hospital Biomedical Research Institute, Seoul 03080, Korea; 3 Department of Obstetrics and Gynecology, Seoul Metropolitan Government Seoul National University Boramae Medical Center, Seoul 07061, Korea; coolluck1979@gmail.com (T.H.K.); tslee70@gmail.com (T.S.L.); ljhw@snu.ac.kr (H.W.J.); 4 Cancer Research Institute, Seoul National University College of Medicine, Seoul 03080, Korea

**Keywords:** cervical cancer, radical hysterectomy, minimally invasive surgery, radiotherapy, adjuvant treatment, recurrence

## Abstract

This study aimed to investigate the impact of adjuvant radiotherapy (RT) on survival outcomes in patients with intermediate-risk, early-stage cervical cancer who underwent radical hysterectomy (RH). From the cervical cancer cohorts of two tertiary hospitals, patients with 2009 FIGO stage IB-IIA who underwent primary RH between 2010 and 2018 were identified. Patients with intermediate-risk factors that met the Sedlis criteria were included. Survival outcomes were compared between the patients who received adjuvant RT (study group; *n* = 53) and those who did not receive adjuvant treatment (control group; *n* = 30). Compared to the control group, the study group showed significantly better recurrence-free survival (RFS; 5-year survival rate, 85.6% vs. 61.0%; *p* = 0.009). In multivariate analysis, adjuvant RT was associated with a significantly lower risk of disease recurrence (adjusted HR, 0.241; 95% CI, 0.082–0.709; *p* = 0.010). In a subgroup that underwent open RH (*n* = 33), adjuvant RT showed a trend toward improved RFS with borderline statistical significance (adjusted HR, 0.098; 95% CI, 0.009–1.027; *p* = 0.053). However, in a subgroup of minimally invasive surgery (*n* = 50), adjuvant RT did not improve RFS. In conclusion, implementation of adjuvant RT significantly reduced the disease recurrence rate in patients with intermediate-risk, stage IB-IIA cervical cancer treated primarily with surgery. Survival benefit from adjuvant RT differed according to the surgical approach.

## 1. Introduction

Cervical cancer is a global burden, ranking fourth for both incidence and mortality among cancers in women, with an estimated 570,000 new cases and 311,000 cancer-related deaths worldwide in 2018 [1]. Owing to disease-specific symptoms and effective screening programs, cervical cancer tends to be diagnosed at an early stage. In the United States, 44% of newly diagnosed cervical cancer patients had localized disease, showing a 91.8% 5-year survival rate [2]. In Korea, where cervical cancer is more prevalent than the Western countries, more than half (55.8%) of the cervical cancer cases were initially found to be confined to the primary site and had a 93.7% 5-year survival rate [3].

Such excellent survival outcomes might be a result of the well-established primary treatment strategies. For treatment of early-stage cervical cancer, primary radical hysterectomy (RH) plus pelvic lymphadenectomy is one of the treatment options [4,5,6,7]. After RH, adjuvant pelvic radiotherapy (RT) is not only recommended in patients with high-risk factors, but also in those who have a combination of the following intermediate-risk factors [6,7]: large tumor size, deep stromal invasion, and lymphovascular space invasion (LVSI), based on a randomized clinical trial (RCT) of Sedlis et al. [8]. In this trial, GOG-92, adjuvant RT following RH significantly reduced the disease recurrence in intermediate-risk, International Federation of Gynecology and Obstetrics (FIGO) stage IB cervical cancer, compared to no further therapy after surgery [8]. Therefore, proficient surgery with additional RT in selected patients is the cornerstone of cervical cancer management. However, whether adjuvant RT has been performed adequately in accordance with the Sedlis criteria and practice guidelines is not known in the real-world clinical practice.

Recently, a phase III RCT, the Laparoscopic Approach to Carcinoma of the Cervix (LACC) trial, reported that minimally invasive surgery (MIS) RH was associated with higher disease recurrence rate and mortality in early-stage cervical cancer, compared to conventional open RH [9]. MIS RH was also associated with worse overall survival (OS) than open RH in a study using two large cancer registries in the United States [10], but no such differences in OS were obseorved between MIS and open RH in subsequent retrospective studies [11,12,13,14]. Depending on the differences in study designs and populations, the latter four studies reported a worse recurrence-free survival (RFS) in patients who underwent MIS RH. However, when stratified by tumor size, inconsistent results were observed such that MIS RH was not a poor prognostic factor among those with stage IB1 and tumor size ≤ 2 cm on preoperative magnetic resonance imaging (MRI) scan [12]. In contrast, in patients with tumor size ≤ 2 cm on final pathology [13] and in low-risk patients with tumor size less than 2 cm without adjuvant treatment [14], MIS RH was a poor prognostic factor. Further research regarding the impact of surgical approach on survival benefit due to adjuvant RT is warranted for intermediate-risk, early-stage cervical cancer.

Herein, our study aimed to question whether the surgical approach influenced the survival benefit achieved from ajduvnat RT in patients with intermediate-risk, early-stage cervical cancer. We compared the survival outcomes between patients who received adjuvant RT and those who did not, considering the surgical approach. In addition, the guideline adherence rate of adjuvant RT in this population was also investigated.

## 2. Materials and Methods

### 2.1. Study Population

This two-institutional retrospective cohort study was approved by the Institutional Review Boards of Seoul National University Hospital (SNUH; No. J-1911-003-1074) and Seoul National University Boramae Medical Center (SNUBMC; No. 20190213/20-2019-7/032). The need for a written informed consent was waived.

We identified patients with 2009 FIGO stage IB-IIA who underwent primary Type C RH at SNUH and SNUBMC between January 2010 and December 2018 [15,16]. Only patients with squamous cell carcinoma, usual type adenocarcinoma, and adenosquamous carcinoma were included in this study. Meanwhile, patients were excluded if: (1) the surgery was performed by inexperienced surgeons (i.e., fellows); (2) they received neoadjuvant chemotherapy before surgery; and (3) they were lost to follow-up during primary treatment or had insufficient clincopathologic data. Based on these criteria, 283 patients were selected.

For the study purpose, we excluded 74 high-risk patients who had at least one of the following three risk factors: pathologically proven lymph node metastasis, resection margin involvement, and parametrial invasion. Finally, we selected only those who met the following Sedlis criteria: (1) positive LVSI and deep third stromal invasion; (2) positive LVSI, middle third stromal invasion, and cervical tumor size ≥ 2 cm; (3) positive LVSI, superficial third stromal invasion, and cervical tumor size ≥ 5 cm; and (4) negative LVSI, middle or deep third stromal invasion, and cervical tumor size ≥ 4 cm [8]. Consequently, 83 intermediate-risk patients were included in the study.

### 2.2. Data Collection

From the medical records, we retrieved the clinicopathologic information of the patients, such as age, conization, histologic type, FIGO stage, pelvic and para-aortic lymphadenectomy, and specific adjuvant treatment. In this study, both clinical and pathologic cervical tumor sizes were collected. In usual cases, clinical tumor size was measured by clinical palpation or inspection. In some cases, we retrospectively measured the tumor size on the preoperative MRI scan and regarded the measured value as clinical tumor size. The pathologic cervical tumor size was used to verify that an individual meets the Sedlis criteria.

Type C RH was performed by eight faculties from the two hospitals, and all of them had completed the fellowship training in gynecologic oncology. After RH, adjuvant RT was recommended to patients with intermediate-risk, early-stage cervical cancer as per the National Comprehensive Cancer Network (NCCN) and Korean Society of Gynecologic Oncology (KSGO) practice guidelines [6,7]. In this study, external beam RT (EBRT) 50.6 Gy in 28 fractions was conducted as adjuvant RT. Because patients with para-aortic lymph node metastasis and/or parametrial invasion were excluded from this study, both extended field RT up to the para-aortic lymphatics and parametrial boost were not performed. Some patients received only EBRT without chemotherapy, while others received a 40 mg/m^2^ of cisplatin weekly for 4–6 cycles during EBRT as concurrent chemoradiotherapy (CCRT).

After the primary treatment, all patients underwent surveillance consisting of computed tomography scans every three–four months for the first two years, every six months for the next two years, and annually thereafter. Based on the Response Evaluation Criteria in Solid Tumors (RECIST) version 1.1, we determined disease progression/recurrence [17]. For the survival analyses, RFS and OS were defined as time intervals from the primary RH to the date of disease recurrence and to the date of death or the end of the study, respectively.

### 2.3. Statistical Analysis

For comparing the clinicopathologic characteristics of the patients, we used Student’s *t*- or Mann-Whitney U-test for continuous variables, and Pearson’s chi-squared or Fisher’s exact test for categorical variables. Survival analyses were performed using the Kaplan–Meier method with the log-rank test. For multivariate analyses, Cox proportional hazards regression models were constructed, and adjusted hazard ratios (aHRs) and 95% confidence intervals (CIs) were calculated. Subgroup analyses were performed based on the surgical approach. Statistical analyses were performed using SPSS statistical software (version 25.0; SPSS Inc., Chicago, IL, USA). GraphPad Prism 5 software (GraphPad Inc., La Jolla, CA, USA) was used to calculate the Pearson’s correlation coefficient test. A *p* < 0.05 was considered statistically significant.

## 3. Results

**Figure 1:**
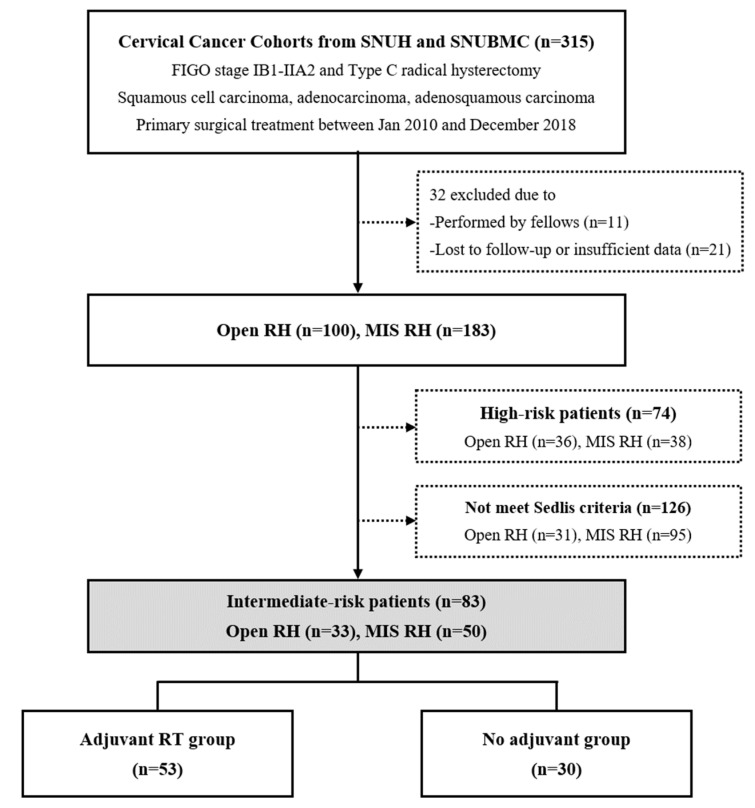
Flow diagrams depicting selection of the study population.

The selection of the study population is presented in Figure 1. Among the 83 surgically treated patients with intermediate-risk, early-stage cervical cancer, 53 received adjuvant RT in accordance with the practice guidelines (study group), while 30 patients did not receive any adjuvant treatment (control group). Therefore, the guideline adherence rate was calculated as 63.9% in this study.

### 3.1. Analysis in All Patients

Table 1 presents the clinicopathologic characteristics of 83 patients with intermediate-risk cervical cancer. The study and control groups showed similar mean age, conization rate, surgical approach, and 2009 FIGO stage. However, patients who received adjuvant RT had higher proportions of squamous cell carcinoma histologic type (90.6% vs. 70.0%; *p* = 0.016) and larger clinical cervical tumor size (mean, 34.8 vs. 25.7 mm; *p* = 0.024), compared to those without any adjuvant treatment. All patients underwent pelvic lymph node dissection with a similar proportion of para-aortic lymphadenectomy between the two groups (20.8% vs. 23.3%; *p* = 0.784). On pathologic examination, no difference in LVSI was observed between the two groups (*p* = 0.151), while deep stromal invasion was more frequently observed in the study group than in the control group (*p* = 0.001). Of the 53 patients in the study group, 11 (20.8%) and 42 (79.2%) received EBRT only and CCRT as adjuvant treatment, respectively.

**Figure 2:**
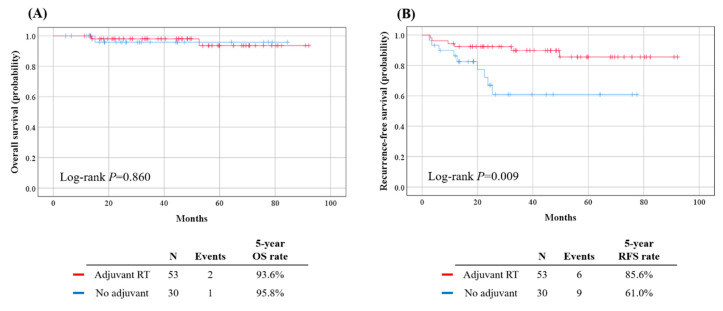
Comparisons of survival outcomes between the study and control groups. (**A**) Overall survival; (**B**) Recurrence-free survival.

During a median follow-up of 40.4 months, there were 15 and 3 cases of recurrence and death, respectively. Both the study and control groups showed similar OS (*p* = 0.860), while the study group showed significantly better RFS (5-year RFS rate, 85.6% vs. 61.0%; *p* = 0.009) (Figure 2).

In multivariate analysis adjusting clinicopathologic variables, adjuvant RT was found to be associated with a significantly lower risk of disease recurrence (aHR, 0.241; 95% CI, 0.082–0.709; *p* = 0.010) (Table 2). In an additional multivariate analysis, after excluding 11 patients who received adjuvant RT only, compared with patients who received no adjuvant treatment, adjuvant CCRT was identified as a favorable prognostic factor for RFS (aHR, 0.129; 95% CI, 0.032–0.516; *p* = 0.004).

### 3.2. Subgroup Analyses by Surgical Approach

Next, we performed subgroup analyses according to the surgical approach used. In a subgroup of patients who underwent open RH (*n* = 33), those who received adjuvant RT and those who did not receive any adjuvant treatment showed similar clinicopathologic characteristics (Table S1). In contrast, in a subgroup of patients who underwent MIS RH (*n* = 50), those who received adjuvant RT had a lower conization rate, higher proportion of squamous cell carcinoma, and deep stromal invasion, compared with those without any adjuvant treatment. Although the pathologic tumor size did not dffer between the two groups, the study group showed a significantly larger clinical cervical tumor size (mean, 34.8 vs. 19.2 mm; *p* = 0.001) (Table S1).

**Figure 3:**
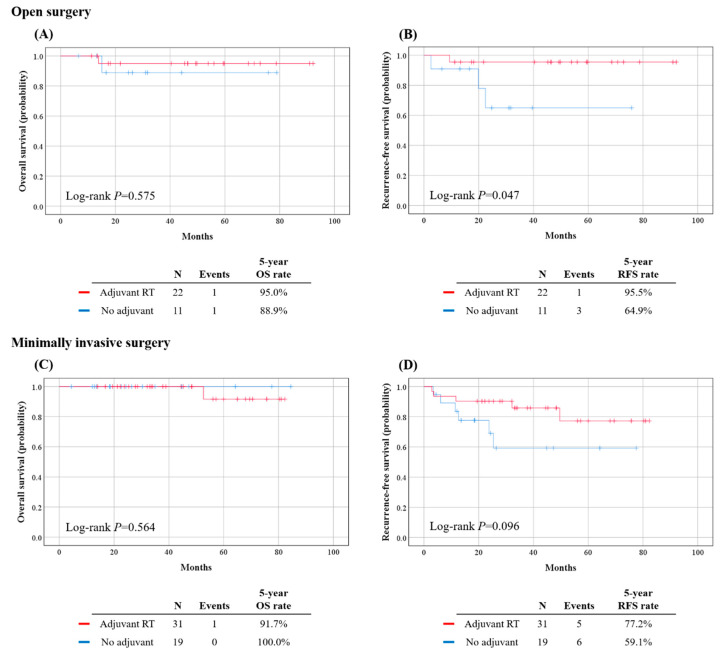
Survival outcomes according to surgical approach of radical hysterectomy. (Upper) Open surgery; (Lower) Minimally invasive surgery. (**A**,**C**) Overall survival; (**B**,**D**) Recurrence-free survival.

Survival analyses of the open RH subgroup showed no difference in OS between the study and control groups (*p* = 0.575); however, a significantly lower recurrence rate was observed in the study group than in the control group (5-year RFS rate, 95.5% vs. 64.9%; *p* = 0.047) (Figure 3A,B). In multivariate analysis, adjuvant RT showed a trend toward improved RFS with borderline statistical significance (adjusted HR, 0.098; 95% CI, 0.009–1.027; *p* = 0.053) (Table 3).

In the MIS RH subgroup, the study and control groups showed similar OS (*p* = 0.564) and RFS (*p* = 0.096) (Figure 3C,D). Multivariate analysis revealed that adjuvant RT did not improve RFS (aHR, 0.305; 95% CI, 0.077–1.214; *p* = 0.092) (Table 3).

### 3.3. Recurrence Patterns and Final Status of Recurred Patients

Recurrence patterns and final status of patients with recurrence are presented in Table S2. In the open RH subgroup (*n* = 33), there was one pelvic recurrence in each group. Among the patients who did not receive any adjuvant treatment, two experienced pelvic recurrence with distant metastasis. In the subgroup of MIS RH (*n* = 50), one central recurrence, one pelvic recurrence, and two cases of lung metastasis were observed among the 31 patients who received adjuvant RT. All four cases were salvaged after the second-line treatment. However, one patient who experienced pelvic recurrence with distant metastasis died despite subsequent treatment. Among the 19 patients who did not receive any adjuvant treatment, two patients had central recurrence, one pelvic recurrence, and one peritoneal recurrence. Two patients experienced both pelvic and distant site recurrences. All six patients were alive after the subsequent treatment.

### 3.4. Guideline Adherence Rate

Each gynecologic oncologist’s adherenece rate to the NCCN and KSGO practice guidelines in intermediate-risk cervical cancer is presented in Table S3. The guideline adherence rate was different depending on the gynecologic oncologist: it ranged from 18.8% to 100.0%. We investigated the correlation between the guideline adherence rate and MIS RH rate, and observed that there was no correlation between the two rates (Pearson’s correlation coefficient *r* = −0.283; *p* = 0.497) (Figure S1).

## 4. Discussion

In this study, we investigated the guideline adherence rate in context of adjuvant RT and survival outcome of intermediate-risk, FIGO stage IB-IIA cervical cancer patients who underwent primary type C RH. Overall, the guideline adherence rate was 63.9% (53/83 patients). While there was no difference in the OS, adjuvant RT was associated with significantly better RFS compared with no further treatment after RH. Moreover, in the subgroup that underwent open RH, adjuvant RT showed a trend toward improved RFS, but statistical significance was not observed. However, in the subgroup that underwent MIS RH, adjuvant did not improve RFS. Our study results demonstrate that survival benefit from adjuvant RT might differ according to the surgical approach.

The GOG-92 study was a landmark study that reported an association between adjuvant RT following RH and a significant decrease in recurrence in patients with node-negative, margin-negative, parametria-negative, early-stage cervical cancer [8]. Since then, the Sedlis criteria have been widely used in identifying intermediate-risk groups, both in clinical practice and in designing clinical trials. Meanwhile, some researchers have suggested other criteria to implement adjuvant RT in the same population. For example, Ryu et al. identified intermediate-risk cervical cancer patients differentially, with their “four-factor model” consisting of tumor size ≥3 cm, deep stromal invasion of the outer third of the cervix, LVSI, and adenocarcinoma or adenosquamous carcinoma histology [18]. The current NCCN guidelines also mentioned that the risk factors may not be limited to the Sedlis criteria [6]. However, the two institutions in the current study adopted the Sedlis critiera in determining whether the patients with node-negative, margin-negative, parametria-negative, early-stage cervical cancer need to undergo adjuvant RT or not after surgery, in accordance with practice guidelines from the NCCN and KSGO [6,7]. Nevertheless, we observed that actual guideline adherence rate at the two institutions was only 63.9%.

Some might argue that adjuvant treatment might be spared in the intermediate-risk group. In their multi-institutional retrospective study, Cibula et al. concluded that patients with intermediate-risk, stage IB cervical cancer had excellent oncological outcomes after type B-C2 RH alone. No difference in the recurrence rate and OS was observed between the patients who received adjuvant RT and those who did not [19]. Another retrospective study on patients with intermediate-risk, stage IB cervical cancer reported similar loco-regional RFS between the groups with or without adjuvant RT after Type C2 RH [20]. These findings are quite different from ours in that we observed significantly better RFS in patients who received adjuvant RT. Such inconsistency may be due to the differences in the study population (e.g., FIGO stage and type of RH). Most of all, the proportion of patients that underwent MIS RH were different among the studies: 16.9% [19], 0.0% [20], and 60.2% (present study). In contrast to the previous studies, we included surgical approach as a covariate in the multivariate analyses and conducted subgroup analyses according to the surgical approach.

Interestingly, the 5-year RFS rate of patients who did not receive any adjuvant treatment was 61.0%. Even if only patients with stage IB disease were considered, the 5-year RFS rate of this group (57.9%) was quite low compared with the two previous studies; 95.7% [19] and 86.6% [20]. The superior disease recurrence rate in the current study might have been due to the adverse effects of MIS RH on survival outcomes that were observed in the LACC trial and other studies [9,10,11,12,13,14]. Specifically, the 5-year RFS rate of the no adjuvant treatment group was 59.1% among patients who underwent MIS RH. However, the 5-year OS rates of the patients who did not receive any adjuvant treatment were similar (94.5% [19], 90.0% [20], and 95.8% (present study)), suggesting that the patients with recurrence were successfully salvaged by the subsequent treatment.

The primary therapeutic effect of adjuvant RT in intermediate-risk patients seems to depend on whether the surgery performed was open surgery or MIS. In subgroup analyses, we observed that the difference in RFS between the groups with and without adjuvant RT changed according to the surgical approach. Among the patients who underwent open RH, adjuvant RT improved RFS compared to no adjuvant treatment. However, among the patients who underwent MIS RH, adjuvant RT did not improve RFS, suggesting that the detrimental effect of MIS nullify the benefit of additional RT. Intraperitoneal tumor spillage and dissemination, which occurs due to MIS-specific conditions such as the use of a uterine manipulator, intracorporeal colpotomy, and CO_2_ penumoperitoneum [21,22], were not overcome by adjuvant RT. For patients undergoing MIS RH, development of a more effective adjuvant treatment strategy is necessary. For example, the addition of immune checkpoint inhibitors to adjuvant RT might be a promising strategy; however, further research is warranted in this regard.

Currently, there is no consensus that adjuvant CCRT is more effective than adjuvant RT only in patients with intermediate-risk, early-stage cervical cancer. According to a recent retrospective study with similar study populations in our study, both 5-year RFS and OS rates were not improved by adding chemotherapy to adjuvant RT compared to adjuvant RT alone [23]. In our study, 79.2% of the adjuvant RT group received CCRT. However, because of the small sample size, we could not conduct further analyses to check the effectiveness of CCRT compared to adjuvant RT alone. An ongoing phase III RCT, GOG-263, comparing the benefit of adjuvant CCRT versus RT alone in patients with intermediate-risk, stage I-II cervical cancer that underwent surgery primarily, will effectively address this issue (NCT01101451).

Our study had several limitations. First, selection bias, which is inevitable in a retrospective study, and small sample size of the study population, which hinders further investations, was the most problematic. For example, it was difficult to ascertain the prognostic impact of chemotherapy on the study group. Second, we only focused on survival outcomes; radiation-related adverse events or quality of life outcomes were not investigated. Finally, a longer follow-up period is needed, as death events were relatively small compared to recurrence. Nevertheless, this study is the first to investigate the survival benefits of adjuvant RT in intermediate-risk, early-stage cervical cancer concerning the surgical approach.

## 5. Conclusions

In conclusion, this two-institutional retrospective cohort study indicated that adjuvant RT significantly reduces disease recurrence rate compared to no adjuvant treatment in patients with intermediate-risk, FIGO stage IB-IIA cervical cancer that were treated primarily with surgery. The survival benefit from adjuvant RT was not observed among patients who underwent MIS RH. The guideline adherence rate regarding the implementation of adjuvant RT varied among gynecologic oncologists. Considering the poor RFS in the subgroup that underwent MIS RH, a more effective adjuvant treatment strategy should be developed. Further studies with a larger sample size are warranted to validate our findings.

## Figures and Tables

**Table 1:** Patients’ clinicopathologic characteristics.

Variables	Adjuvant Radiotherapy (*n* = 53, %)	No Adjuvant Treatment (*n* = 30, %)	*p*
Age, years			
Mean ± SD	51.6 ± 11.5	53.2 ± 14.2	0.587
Surgical approach			0.776
Open	22 (41.5)	11 (36.7)	
Laparoscopy	28 (52.8)	18 (60.0)	
Robot-assisted surgery	3 (5.7)	1 (3.3)	
Conization	9 (17.0)	10 (33.3)	0.088
Histologic type			0.006
Squamous cell carcinoma	48 (90.6)	21 (70.0)	
Adenocarcinoma	1 (1.9)	7 (23.3)	
Adenosquamous carcinoma	4 (7.5)	2 (6.7)	
2009 FIGO stage			0.375
IB1	28 (52.8)	20 (66.7)	
IB2	13 (24.5)	7 (23.3)	
IIA1	4 (7.5)	2 (6.7)	
IIA2	8 (15.1)	1 (3.3)	
Para-aortic LN sampling/dissection	11 (20.8)	7 (23.3)	0.784
Clinical cervical tumor size, mm			
Mean ± SD	34.8 ± 17.0	25.7 ± 17.5	0.024
Pathologic cervical tumor size, mm			
Mean ± SD	50.5 ± 18.1	45.8 ± 12.6	0.219
<20	1 (1.9)	1 (3.3)	0.976
≥20 and <40	14 (26.4)	8 (26.7)	
≥40 and <50	10 (18.9)	6 (20.0)	
≥50	28 (52.8)	15 (50.0)	
LVSI	35 (66.0)	15 (50.0)	0.151
Stromal invasion			0.001
Superficial 1/3	1 (1.9)	1 (3.3)	
Middle 1/3	4 (7.5)	12 (40.0)	
Deep 1/3	48 (90.6)	17 (56.7)	
Sedlis criteria			0.034
LVSI (+) Deep 1/3, Tumor size any	33 (62.3)	10 (33.3)	
LVSI(+) Middle 1/3, Tumor ≥ 20 mm	1 (1.9)	4 (13.3)	
LVSI(+) Superficial 1/3, Tumor ≥ 50 mm	1 (1.9)	1 (3.3)	
LVSI(-), Middle or deep 1/3, Tumor ≥ 40 mm	18 (34.0)	15 (50.0)	
Adjuvant treatment			N/A
RT only	11 (20.8)	0	
CCRT	42 (79.2)	0	

Presented with mean ± SD (range) or *n* (%). Abbreviations: CCRT, concurrent chemoradiation therapy; FIGO, International Federation of Gynecology and Obstetrics; LN, lymph node; LVSI, lymphovascular space invasion; RT, radiotherapy; SD, standard deviation; N/A, not applicable.

**Table 2:** Factors associated with recurrence-free survival.

Variables		Univariate Analysis			Multivariate Analysis		
		HR	95% CI	*p*	aHR	95% CI	*p*
Age, years	≥50 vs. <50	0.558	0.198–1.573	0.270	0.427	0.144–1.270	0.126
Pre-operative conization	Yes vs. No	0.867	0.244–3.076	0.825			
Histologic type	Non-SCC vs. SCC	0.826	0.186–3.679	0.802	0.453	0.096–2.137	0.317
2009 FIGO stage	IB2-IIA2 vs. IB1	0.715	0.244–2.093	0.541	0.738	0.236–2.301	0.600
Cervical tumor size, mm	Pathologic, ≥40 vs. <40	0.896	0.306–2.623	0.841			
Deep stromal invasion	Yes vs. No	0.416	0.141–1.221	0.110			
Surgical approach	MIS vs. Open	1.868	0.594–5.874	0.285	1.730	0.532–5.626	0.362
Adjuvant treatment	RT vs. No adjuvant	0.268	0.094–0.766	0.014	0.241	0.082–0.709	0.010

Abbreviations: aHR, adjusted hazard ratio; CI, confidence interval; FIGO, International Federation of Gynecology and Obstetrics; HR, hazard ratio; MIS, minimally invasive surgery; RH, radical hysterectomy; RT, radiotherapy; SCC, squamous cell carcinoma.

**Table 3:** Factors associated with recurrence-free survival according to surgical approach.

Variables		Open RH (*n* = 33)			MIS RH (*n* = 50)		
		aHR	95% CI	*p*	aHR	95% CI	*p*
Age, years	≥50 vs. <50	0.176	0.015–2.060	0.166	0.621	0.178–2.166	0.455
Histologic type	Non-SCC vs. SCC				0.851	0.171–4.228	0.844
2009 FIGO stage	IB2-IIA2 vs. IB1	0.377	0.033–4.346	0.434			
Cervical tumor size, mm	Clinical, ≥40 vs. <40				1.648	0.354–7.681	0.525
Deep stromal invasion	Yes vs. No	0.102	0.004–2.954	0.184			
Adjuvant treatment	RT vs. No adjuvant	0.098	0.009–1.027	0.053	0.305	0.077–1.214	0.092

Abbreviations: aHR, adjusted HR; CI, confidence interval; FIGO, International Federation of Gynecology and Obstetrics; HR, hazard ratio; MIS, minimally invasive surgery; RH, radical hysterectomy; RT, radiotherapy; SCC, squamous cell carcinoma.

## Supplementary Materials

The following are available online at https://www.mdpi.com/2077-0383/9/11/3545/s1, Figure S1: Correlation between the guideline adherence rate and MIS RH rate, Table S1: Patients’ clinicopathologic characteristics according to surgical approach, Table S2: Recurrence patterns and final status of recurred patients, Table S3: Minimally invasive surgery rate and guideline adherence rate by gynecologic oncologists.
