# Supplementary material for: Impact of Adjuvant Radiotherapy on Survival Outcomes in Intermediate-Risk, Early-Stage Cervical Cancer: Analyses Regarding Surgical Approach of Radical Hysterectomy
Source: J Clin Med. 2020 Nov 3;9(11):3545. doi: 10.3390/jcm9113545 (PMC7692216; doi:10.3390/jcm9113545)
Supplement: Supplementary file 1 [file jcm-09-03545-s001.zip › Table S1.docx]

**Table S1.** Patients’ clinicopathologic characteristics according to surgical approach.

| **Variables** | **Open RH (*n*=33)** | | | **MIS RH (*n*=50)** | | |
| --- | --- | --- | --- | --- | --- | --- |
|  | **Adjuvant radiotherapy**  **(*n*=22, %)** | **No adjuvant treatment**  **(*n*=11, %)** | ***p*** | **Adjuvant radiotherapy**  **(*n*=31, %)** | **No adjuvant treatment**  **(*n*=19, %)** | ***p*** |
| Age, years |  |  |  |  |  |  |
| Mean ± SD | 49.0 ± 11.7 | 57.5 ± 15.3 | 0.086 | 53.5 ± 11.2 | 50.7 ± 13.3 | 0.428 |
| Conization | 4 (18.2) | 1 (9.1) | 0.643 | 5 (16.1) | 9 (47.4) | 0.017 |
| Histologic type |  |  | 0.142 |  |  | 0.027 |
| Squamous cell carcinoma | 20 (90.9) | 8 (72.7) |  | 28 (90.3) | 13 (68.4) |  |
| Adenocarcinoma | 1 (4.5) | 3 (27.3) |  | 0 | 4 (21.1) |  |
| Adenosquamous carcinoma | 1 (4.5) | 0 |  | 3 (9.7)) | 2 (10.5) |  |
| 2009 FIGO stage |  |  | 0.096 |  |  | 0.206 |
| IB1 | 10 (45.5) | 4 (36.4) |  | 18 (58.1) | 16 (84.2) |  |
| IB2 | 4 (18.2) | 6 (54.5) |  | 9 (29.0) | 1 (5.3) |  |
| IIA1 | 2 (9.1) | 1 (9.1) |  | 2 (6.5) | 1 (5.3) |  |
| IIA2 | 6 (27.3) | 0 |  | 2 (6.5) | 1 (5.3) |  |
| Para-aortic LN sampling/dissection | 6 (27.3) | 3 (27.3) | >0.999 | 5 (16.1) | 4 (21.1) | 0.715 |
| Clinical cervical mass size, mm |  |  |  |  |  |  |
| Mean ± SD | 34.7 ± 19.5 | 37.0 ± 16.0 | 0.736 | 34.8 ± 15.4 | 19.2 ± 15.2 | 0.001 |
| Pathologic cervical tumor size, mm |  |  |  |  |  |  |
| Mean ± SD | 52.0 ± 21.2 | 47.7 ± 9.0 | 0.429 | 49.4 ± 15.8 | 44.7 ± 14.4 | 0.301 |
| <20 | 1 (4.5) | 0 | 0.682 | 0 | 1 (5.3) | 0.555 |
| ≥20 and <40 | 6 (27.3) | 2 (18.2) |  | 8 (25.8) | 6 (31.6) |  |
| ≥40 and <50 | 3 (13.6) | 3 (27.3) |  | 7 (22.6) | 3 (15.8) |  |
| ≥50 | 12 (54.5) | 6 (54.5) |  | 16 (51.6) | 9 (47.4) |  |
| LVSI | 17 (77.3) | 6 (54.5) | 0.240 | 18 (58.1) | 9 (47.4) | 0.461 |
| Stromal invasion |  |  | 0.304 |  |  | 0.002 |
| Superficial 1/3 | 0 | 0 |  | 1 (3.2) | 1 (5.3) |  |
| Middle 1/3 | 2 (9.1) | 3 (27.3) |  | 2 (6.5) | 9 (47.4) |  |
| Deep 1/3 | 20 (90.9) | 8 (72.7) |  | 28 (90.3) | 9 (47.4) |  |
| Sedlis criteria |  |  | 0.113 |  |  | 0.209 |
| LVSI (+) Deep 1/3, Tumor size any | 17 (77.3) | 5 (45.5) |  | 16 (51.6) | 5 (26.3) |  |
| LVSI(+) Middle 1/3, Tumor ≥20 mm | 0 | 1 (9.1) |  | 1 (3.2) | 3 (15.8) |  |
| LVSI(+) Superficial 1/3, Tumor ≥50 mm | 0 | 0 |  | 1 (3.2) | 1 (5.3) |  |
| LVSI(-), Middle or deep 1/3, Tumor ≥40 mm | 5 (22.7) | 5 (45.5) |  | 13 (41.9) | 10 (52.6) |  |
| Adjuvant treatment |  |  | N/A |  |  | N/A |
| RT only | 4 (18.2) | 0 |  | 7 (22.6) | 0 |  |
| CCRT | 18 (81.8) | 0 |  | 24 (77.4) | 0 |  |

Presented with mean ± SD (range) or *n* (%). Abbreviations: CCRT, concurrent chemoradiation therapy; FIGO, International Federation of Gynecology and Obstetrics; LN, lymph node; LVSI, lymphovascular space invasion; RT, radiotherapy; SD, standard deviation; N/A, not applicable.
