# Supplementary material for: Impact of Adjuvant Radiotherapy on Survival Outcomes in Intermediate-Risk, Early-Stage Cervical Cancer: Analyses Regarding Surgical Approach of Radical Hysterectomy
Source: J Clin Med. 2020 Nov 3;9(11):3545. doi: 10.3390/jcm9113545 (PMC7692216; doi:10.3390/jcm9113545)
Supplement: Supplementary file 1 [file jcm-09-03545-s001.zip › Table S3.docx]

| **Table S3.** Minimally invasive surgery rate and guideline adherence rate by gynecologic oncologists | | | | | | | | | |
| --- | --- | --- | --- | --- | --- | --- | --- | --- | --- |
| **Gynecologic oncologist** | **#1**  **(*n*=15, %)** | **#2**  **(*n*=10, %)** | **#3**  **(*n*=19, %)** | **#4**  **(*n*=5, %)** | **#5**  **(*n*=8, %)** | **#6**  **(*n*=5, %)** | **#7**  **(*n*=5, %)** | **#8**  **(*n*=16, %)** | **Total**  **(*n*=83, %)** |
| MIS RH | 12 (80.0) | 0 (0) | 17 (89.5) | 1 (20.0) | 7 (87.5) | 4 (80.0) | 1 (20.0) | 8 (50.0) | 50 (60.2) |
| Guideline adherence | 14 (93.3) | 9 (90.0) | 12 (63.2) | 5 (100.0) | 3 (37.5) | 4 (80.0) | 3 (60.0) | 3 (18.8) | 53 (63.9) |
| Abbreviations: MIS, minimally invasive surgery; RH, radical hysterectomy. | | | | | | | | | |
