# Supplementary material for: Impact of Adjuvant Radiotherapy on Survival Outcomes in Intermediate-Risk, Early-Stage Cervical Cancer: Analyses Regarding Surgical Approach of Radical Hysterectomy
Source: J Clin Med. 2020 Nov 3;9(11):3545. doi: 10.3390/jcm9113545 (PMC7692216; doi:10.3390/jcm9113545)

**Table S2.** Recurrence patterns and final status of recurred patients

|  | **Open RH (*n*=33)** | | **MIS RH (*n*=50)** | |
| --- | --- | --- | --- | --- |
|  | **Adjuvant radiotherapy**  **(*n*=22)** | **No adjuvant treatment**  **(*n*=11, %)** | **Adjuvant radiotherapy**  **(*n*=31)** | **No adjuvant treatment**  **(*n*=19)** |
| Recurrence patterns |  |  |  |  |
| Central | 0 | 0 | 1 | 2 |
| Pelvis | 1 (1 death) | 1 | 1 | 1 |
| Lung only | 0 | 0 | 2 | 0 |
| Peritoneum | 0 | 0 | 0 | 1 |
| Pelvis plus distant site | 0 | 2 (1 death) | 1 (1 death) | 2 |
| Total recurrence | 1 | 3 | 5 | 6 |
| Death from disease | 1 | 1 | 1 | 0 |
| Abbreviations: MIS, minimally invasive surgery; RH, radical hysterectomy. | | | | |


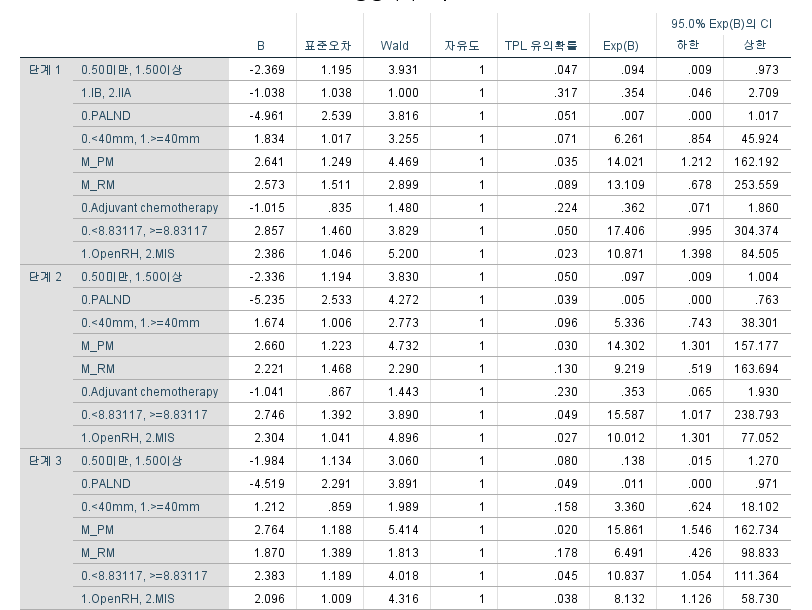


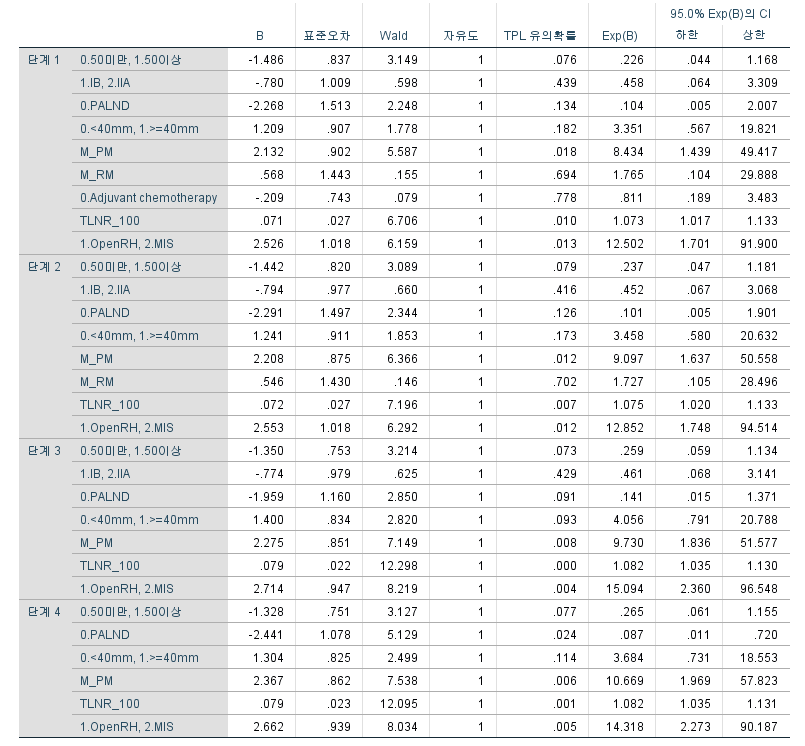

Supplement: Supplementary file 1 [file jcm-09-03545-s001.zip › Table S2.docx]
