# Supplementary figures and images for: Impact of Adjuvant Radiotherapy on Survival Outcomes in Intermediate-Risk, Early-Stage Cervical Cancer: Analyses Regarding Surgical Approach of Radical Hysterectomy
Source: J Clin Med. 2020 Nov 3;9(11):3545. doi: 10.3390/jcm9113545 (PMC7692216; doi:10.3390/jcm9113545)

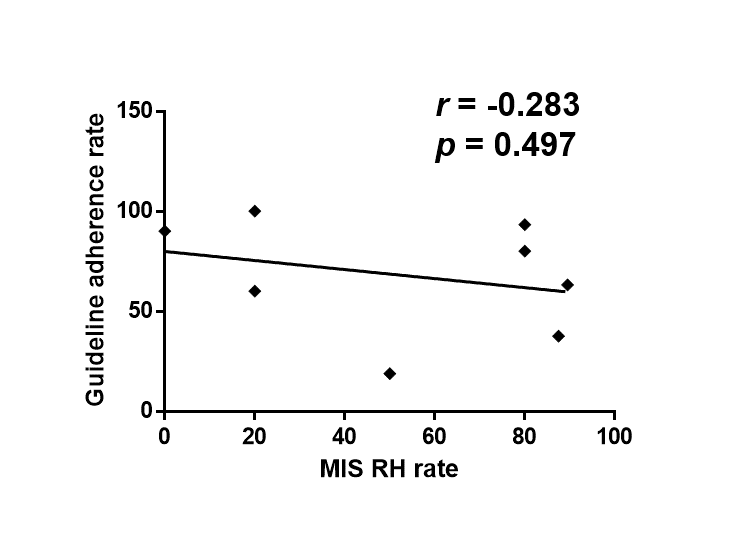

Supplement: Supplementary file 1 [file jcm-09-03545-s001.zip › Figure S1.png]
